# Female sex workers perspectives and concerns regarding HIV self-testing: an exploratory study in Tanzania

**DOI:** 10.1186/s12889-020-09105-6

**Published:** 2020-06-18

**Authors:** Soori Nnko, Daniel Nyato, Evodius Kuringe, Caterina Casalini, Amani Shao, Albert Komba, John Changalucha, Mwita Wambura

**Affiliations:** 1 Department of Sexual and Reproductive Health, National Institute for Medical Research, P.O Box 1462, Mwanza, Tanzania; 2 Sauti Program, Jhpiego Tanzania - an affiliate of Johns Hopkins University, P.O Box 9170, Dar es Salaam, Tanzania

**Keywords:** HIV self-testing, Female sex workers, acceptability, exploratory study, Tanzania

## Abstract

****Background**:**

HIV testing is a gateway to HIV care and treatment for people diagnosed with HIV and can link those with negative results to HIV preventive services. Despite the importance of HIV testing services (HTS) in HIV control, uptake of HTS among female sex workers (FSWs) across sub-Saharan Africa (SSA) remains sub-optimal. Concerns about stigma associated with sex work and fear of loss of livelihood if HIV status becomes known, are some of the restrictions for FSWs to utilize HTS offered through health care facilities. Introduction of HIV self-testing (HIVST) may mitigate some of the barriers for the uptake of HTS. This study explored the acceptability of FSWs towards the introduction of HIVST in Tanzania.

****Methods**:**

We conducted an exploratory study employing in-depth interviews (IDI) and participatory group discussions (PGD) with FSWs in selected regions of Tanzania. Study participants were recruited through snowball sampling. Data were thematically analysed by two analysts using *NVivo* software. The analysis was informed by the social-ecological model and focused on factors associated with the acceptability of HIVST.

****Results**:**

We conducted 21 PGD sessions involving 227 FSWs. Twenty three IDIs were conducted to complement data collected through PGD. Our study has demonstrated that **FSWs are enthusiastic toward HIVST.** Convenience (time and cost saved), and belief that HIVST will increase privacy and confidentiality motivated participants’ support for the self-testing approach. Participants did express concerns about their ability to interpret and trust the results of the test. Participants also expressed concern that HIVST could cause personal harm, including severe distress and self-harm for individuals with a reactive test. Very likely, concern about adverse effects of HIVST was linked to the study participants’ lay perception that HIVST would be provided only through unassisted modality.

**Conclusions:**

FSWs demonstrated high enthusiasm to use the HIVST once it becomes available. Expectations for increased confidentiality, autonomy, and reduced opportunity costs were among the leading factors that attracted FSWs to HIVST. The major obstacles to the acceptability of HIVST included fear of HIV reactive test and not trusting self-diagnoses. Our findings underscore the importance of providing adequate access to counselling and referral services in conjunction with HIVST.

## Background

Globally, FSWs are at an increased risk of HIV infection compared to women in the general population [1, 2]. According to a systematic review and meta analysis of studies published in 2012, FSWs from low and middle income countries were 13.5 times more likely to be infected with HIV as compared to women in the general population [2]. Pooled HIV prevalence among FSWs from those countries was estimated to be 11.8% [2]. A study conducted in Tanzania in 2013 estimated HIV prevalence in FSWs to be 26.6% [3], compared to HIV prevalence of 6.2% among women of reproductive age (15–49 years) from the general population [4].

Since most new HIV infections are caused by persons unaware of their HIV sero-status [5, 6], HIV testing is a crucial step for getting people into prevention, care and treatment services. A variety of approaches have been employed to deliver HIV testing services (HTS) including; integrating HTS into family planning or antenatal care services [7], outpatient services [8–10] and the use of community-based testing [9, 11–16]. Also, a multitude of community-based HTS approaches such as mobile services in Benin [17], mapping in South Africa [18] and drop-in centres in the Democratic Republic of Congo [19] have explicitly been designed to increase uptake among FSWs. Despite the success of these delivery models, uptake of HTS among FSWs is still suboptimal [20, 21].

FSWs face particular barriers to accessing HTS, including high levels of stigma associated with sex work [22–25] and fear of loss of livelihood if HIV status becomes known [26–28]. High stigma toward sex work and HIV has been documented to cause the reluctance for FSWs to seek family planning services [29] and to initiate and progress into the continuum of care [30]. Thus, more efforts are needed to reach FSWs, proportional to their risk of HIV infection. In 2017, the World Health Organization (WHO) recommended HIV self-testing (HIVST) and partner notification services in countries with a generalized HIV epidemic [31]. Self-testing is thought to be effective service delivery modality for FSWs, particularly to mitigate structural barriers which hinder them from accessing health services in the facility setting. Both supervised/assisted and private/unassisted modalities are available for HIVST and participants are free to choose the option of their interest [32, 33].

Studies exploring factors associated with HIVST have largely compared the acceptability of HIVST across different target populations [34]. Furthermore, most of the studies regarding the acceptability of HIVST among members of key populations (KPs) men who have sex with men (MSM), female sex workers (SW), people who inject drugs (PWID), transgender people, and people in prisons or closed settings have predominantly taken place in clinical trial settings [35]. Studies conducted among FSWs in clinical trial settings have reported high acceptability and accessibility to HIVST [34]. However, little is known about how KPs outside clinical trial settings, and particularly FSWs perceive HIVST, and how they would respond to the introduction of this service. We conducted an exploratory study to inform the formulation of programmatic approaches to deliver comprehensive HIV combination prevention program in Tanzania (Sauti Project) [35]. By the time we conducted our study, HIVST policy had not been introduced in Tanzania, and therefore we wanted to understand the acceptability of HIVST among FSWs outside clinical trial settings.

## Methods

### Theoretical framework

Based on the socio-ecological model (SEM), HIV risk perceptions or and behaviours leading to HIV infections can take place at different levels, including, individual, relational, community or social groups, and national policy (enabling environment) levels [36–38]. However, realising that SEM is flexible and that ‘no one model is sufficient to describe factors that influence individual behaviour across the diverse domain’, Baral and colleagues propose for a “modified social-ecological model” (MSEM) which incorporates the *‘stages of HIV epidemic* [39]. This modification underscores that apart from the fact that HIV infections and transmissions occur in diverse social-cultural contexts, *stages of HIV epidemics* inform not only HIV risk perceptions and behaviour for HIV acquisitions and transmissions [39, 40], but also public discourses and strategies regarding HIV preventions. Although the modified version of SEM was formulated to analyse people’s risk behaviour for HIV infections, we find it to be a relevant framework to understand FSWs’ perspectives toward HTS including HIVST, particularly regarding public discourses on risk perceptions and contribution of Key populations (KPs) in HIV transmissions.

### Program setting

The study was conducted to inform a comprehensive HIV combination prevention program which delivers biomedical, behavioural and structural interventions to FSWs and other key populations in Tanzania (the Sauti project). Sauti is a five-year program funded by PEPFAR through the US Agency for International Development (USAID), administered by Jhpiego- an affiliate of Johns Hopkins University in collaboration with Engender Health, Pact Tanzania and the National Institute for Medical Research (NIMR), under Tanzania’s Ministry of Health, Community Development, Gender, Elderly and Children (MoHCDGEC).

This study was conducted in 2017 in Dar es Salaam city, Iringa, Mbeya and Shinyanga regions, where the Sauti program interventions were being implemented in that year. Dar es Salaam is the largest city in Tanzania located at the eastern part of Tanzania. Iringa and Mbeya regions are in the southern highlands. Mbeya is also bordering Zambia and Malawi. Shinyanga is located in north-western Tanzania, around Lake Victoria. Iringa and Shinyanga regions have tea plantations and mining sites respectively, which attracted a high number of seasonal migrants including FSWs and labour forces. Being the major cities, and hubs for the Tanzania-Zambia highway, Dar es Salaam and Mbeya have a high traffic of trucks transiting between Tanzania and other landlocked countries in southern and central Africa.

Surveys conducted to estimate population sizes of FSWs in the country have shown that Mbeya, Dar es Salaam and Shinyanga are among the regions with the highest number of FSWs in Tanzania [41, 42]. These regions were selected for our study because we anticipated that they would have an optimal number of FSWs to allow us to obtain a sufficient sample size. Since we were also interested to understand FSWs’ sexual behaviour and their HIV risk perceptions, we choose to work in areas with high risks for HIV transmissions. These four regions were deemed to be the most appropriate sites since they have the highest HIV prevalence. With -exception of Dar es Salaam (4.7%), the remaining three regions have HIV prevalence higher than the national average (5%) [43].

### Study design

We conducted a qualitative study utilizing participatory group discussions (PGDs) and in depth interviews (IDI) to capture information on community norms and individual lived experiences respectively. IDI is a suitable method for obtaining sensitive information, which is considered a taboo and should not be discussed in public, whereas PGD is ideal for the collection of information which is perceived to be less sensitive including perceptions towards health interventions.

PGDs were preferred to traditional focus group discussions (FGD), since even though both methods are participatory, participants in PGDs are, in addition to group discussions, involved in many other interactive research activities which generate depth understanding of phenomena, than with the FGDs. In our study, apart from participating in consensual building group discussions, participants were subdivided into smaller groups (involving three to five persons) to brainstorm and draw maps to indicate locations where FSWs could obtain health care services including HTS. During the brainstorming sessions, participants ranked their preferences to each of the identified locations and discussed the extent to which availability of services such as HIVST kits in those sites could facilitate or hinder its utilization.

### Sampling and recruitment of study participants

We conducted IDIs with FSWs from seven wards across the four regions, involving three participants per ward. We designed to continue recruiting new batches of FSWs and interview them until the saturation point was reached. The saturation point was reached after conducting IDIs with 23 FSWs. Eligible participants for the study were females, aged 18 years or above, self-identified as sex workers, consenting to participate in the study. The Sauti program defined a sex worker as someone whose primary source of income is sex work.

Peer referral sampling strategies were used to recruit participants for IDI. Community gatekeepers, including owners of entertainment facilities and Civil Society Organisations (CSOs), provided the research team with the list of three FSWs who constituted the first batch of FSWs to interview. The first batch of participants constituted the most influential and peer leaders of FSWs. After the initial interviews, participants were asked to recruit up to three FSWs from their networks. After the completion of 23 IDIs, we then started to recruit participants for PGD sessions. Similar to the procedure used to recruit participants for IDI, we employed snowball (peer referral) sampling to recruit participants for PGD sessions. A total of 21 PGD sessions were conducted involving 227 participants.

### Data collection

Data collection was conducted by six interviewers experienced in qualitative research methods. Before data collection, the interviewers attended a 2 weeks training, which covered issues related to the principles of research ethics, consenting procedures, confidentiality and techniques to elicit sensitive information through face to face interviews. Also, this training included a practical session to orient researchers to study tools and conduct mock interviews. The study tools included study’ standard operating procedures (SOP), IDI guides and PGD schedules that were developed specifically for this study [44].

A moderator and a note-taker facilitated PGD sessions. Each PGD session involved between eight to twelve participants. Before the IDI and PGD sessions, interviewers collected socio-demographic data from each participant. Given that HIVST was not yet rolled-out when this study was taking place, interviewers read to the participant a description of HIVST before the interview.

IDI and PGD sessions were conducted in private locations, including hired rooms in Guest Houses, or in CSO offices. IDIs and PGDs were conducted in the Kiswahili, a national language widely spoken in Tanzania. Separate consents were provided for participation in IDI and PGD. On average, each IDI took about 45 to 60 min, whereas PGD sessions took about 90 to 120 min. Both the IDI and PGD sessions were audio-recorded.

### Data management and analysis

Data collection and analysis took place concurrently. The field notes were typed onto laptops and together with the audio recordings transferred to NIMR server daily via a secure file transfer protocol. Throughout data collection, once after every 2 days, the senior social scientists (SN & DN) obtained written field notes and debriefings reports from the interviewers. These written notes and verbal reports enabled the senior researchers to identify key themes to facilitate an iterative process of data collection and analysis. Through these consultations, the senior researchers were also able to track if there were protocol deviations or any other inconsistency of data collection. Any noted gaps and discrepancies from data were further explored in the subsequent IDI and PGD sessions. All audio files were transcribed verbatim in Kiswahili and translated into English. The written notes were typed and merged with corresponding transcribed texts.

A cascading process was used to analyze data. In the first stage, a narrative, including verbatim transcripts from each PGD and IDIs, was written. In this narrative, the researchers used a deductive approach to identify themes of interest. The predetermined (a priori*)* coding themes were developed during the study design stage. The final coding themes were developed by combining the predetermined and new ones that emerged during preliminary content analysis Table 1. The second stage of analysis consisted of transferring the compiled data by themes into the qualitative software (NVivo 11), which allowed the identification of regularities and patterns. The conclusions were drawn based on predetermined and emerging themes, regularities, patterns, and causal flows towards study objectives.

**Table 1:** Example of themes, analytical categories and subcategories developed for the data analysis

Domain	Themes (main topics)	Analytical categories	Analytical subcategories
Individual	Participants attributes	Socio-economic attributes	Family backgrounds;
			Level of education; age; residence
			Level of income
	Awareness toward HTS Access to HTS and health care delivery points	Knowledge and perceptions toward HTS and HIVST	Attitude toward HTS
			Awareness about HIVST
Societal			Rumour toward HIVST
Individual			Previous experience to HTS
			HIV risk perceptions
Institutional		Availability of services	Accessibility to delivery points
		Quality of the service	Attitude of health care workers
			Privacy of the client
			Self-determination of clients
Societal	Barriers to HIVST	HIV stigma	Concern about social support
Individual		Self-efficacy	Concern about user’ error
Societal		Social norms toward HIV	Negative narratives about HIV
			Fear about a reactive test

Table 1 presents themes, analytical categories and sub-categories developed for data analysis.

## Results

### Socio-demographic characteristics

A total of 227 FSWs participated in 21 sessions of PGDs. Twenty three of the FSWs who participated in PGD sessions were also interviewed through IDIs. The median age of the study participants was 24 years. Of the 227 participants, 18 (8%) had never been to school, and 117 (51.5%) had completed primary school education. One hundred forty-six participants (64.4%) reported to have engaged in sex work for more than 1 year. Sixty-three per cent of FSWs (143) solicited their clients from recreational facilities (e.g. hotels, bar, disco halls). Table 2 describes the participants’ characteristics.

**Table 2:** Participants socio-demographic characteristics (*n* = 227)

Variable	Median [Range] or N (%)
**Age**	24 [18–42]
**Marital status**	
Single	106 (46.7)
Living with a permanent partner (cohabiting/married)	89 (39.2)
Divorced	24 (10.6)
Widow	8 (3.5)
**Children (living)**	
None	43 (18.9)
1 child	106 (46.7)
≥ 2 children	78 (34.4)
**Education**	
Never attend formal education	18 (8)
Completed some primary school	117 (51.5)
Secondary school	80 (35.2)
Post-secondary education	12 (5.3)
**Duration in sex work**	
Less than 1 year	43 (18.9)
1–3 years	109 (48)
> 3 years	37 (16.4)
Cannot remember	38 (16.7)
**Site where FSW solicited clients**	
Streets / roadsides	48 (21.1)
Recreational facilities	143 (63.1)
Homes or brothels (private venue)	28 (12.3)
Social media (e.g. websites)	8 (3.5)

### Awareness and attitude about HIV testing

Knowledge about the availability of HIV testing and testing practices was generally high, where 93.0% of participants reported having tested for HIV in the past 2 years. Of those who tested, 13.6% reported being HIV positive. Among those who tested HIV negative, 80% were willing to test again. Over half (56.3%) of those who indicated that they would re-test thought that HIV testing services were supposed to be offered only in the health facility setting.

When participants were asked if they have ever heard about HIV self-testing, only 25% reported to know it, among whom none reported to have ever heard of HIVST done using oral fluid.

### Positive viewpoints about HIVST

#### Improved confidentiality, convenience and empowerment

In all PGD sessions, the participants reached the consensus that the introduction of HIVST would be positively received. FSWs thought the availability of HIVST would minimize dependency on health workers, and inconveniences they encounter when seeking services from the health facilities. The belief that HIVST would restore self-autonomy is captured by a remark made by one of the FSW from a PGD session:

*If kits for HIV testing become available at our homes, it’s very easy for us to test when we need it […]. We shall test promptly without any delay. Testing HIV at health facility requires people to make some prior preparations to reach the facility***[PGD_ Iringa]**

In all PGD sessions, the participants reached the consensus that, availability of HIVST can guarantee complete privacy during HIV testing. The view that availability of HIVST would improve privacy was also reported by 17 FSWs, out of the 23 he FSW who participated in IDI.

*Don't you know that if you are tested by someone else s/he will know your status and start telling other people that you are already infected?[...] If they bring kits to us we shall test ourselves and, nobody else will know [results]* [**IDI_30 years_Shinyanga]**

The participants believed that the introduction of HIVST would also help to mitigate stigma and discrimination ascribed to sex work and the people living with HIV.

*People are not trustful. You cannot even trust your siblings. [Your sibling] can inadvertently tell other people that you know our sister is sick. [If HIV status becomes known to other people] that will be the end of your business [...] Remember there is a possibility of being stigmatised.***[IDI_44 years_Mbeya]**

Three FSWs who participated in IDI reported that, they were reluctant to seek HIV test at health facilities because they feared that health workers may disclose their HIV status to other people. According to them the availability of HIVST would help to minimize breach of confidentiality. FSWs who had already visited health facilities for HTS reported that HIV testing involved prolonged biomedical protocol before they receive their test results. These participants believed that the availability of HIVST will help to save the time they would lose to seek HTS.

### Preference toward delivery points and willingness to pay for the services

Participants reported that they would be willing to contribute a modest amount of money to access HIVST. In most PGDs, participants felt that they would be willing to pay a range of Tanzania shillings 2000–3000 [equivalent to USD 1–1.5] per kit. A participant further describes this:

*I wish the price [of HIVST test kit] to be around two to three thousand shillings [...] because we earn about ten thousand shillings from a single client. We could therefore spend three thousand for HIV testing and use the remaining money for the home chores***[IDI_21 years_Shinyanga]**

In all PGDs, participants expressed preference toward HIVST over health facility-based testing. Participants would like to see HIVST kits to be available in nearby pharmacies (highest priority), private health facilities (medium priority) and public health facilities (lowest priority). Alternatively, participants preferred the test kits to be available in informal sites including community spots, guesthouses, public washrooms and pubs, or through community based organisations (CBOs) serving FSWs.

### Negative viewpoints about HIVST

#### Social norms toward sex work and HIV

Adverse social norms and stigmatizing narratives toward HIV and people living with HIV were described as barriers to self-testing. Participants acknowledged their own increased risk for HIV infection due to their involvement in high-risk sexual behaviour. Consequently, FSWs reported fear of self-testing, because they suspected themselves to be already infected with HIV and were not able to cope with the reactive test. Participants from the study, who were concerned about coping with a reactive test, appeared not to be aware of the “assisted” option of HIVST.

Few participants thought the availability of self-testing services would lead to a deliberate spread of HIV. The view that self-testing would create room for malicious FSWs to spread HIV to their clients was raised in two PGDs.

*Most of the women involved in sex work are not safe […]. So, if HIVST becomes available [they will test themselves and, they will never disclose their HIV positive status to their partners***[PGD_Dar es Salaam]**

*If testing is done secretly, there will be an increased spread of HIV, because after a person has discovered to be infected, she will deliberately transmit HIV to other people***[PGD_Iringa]**

#### Fear of social harms

Despite considerable support for HIVST, several considerations were expressed about potential adverse outcomes related to conducting an HIV test without oversight by trained professionals. Four participants from IDIs who were not aware of the availability of the assisted option of HIVST expressed a concern that a reactive test result may cause severe distress to FSWs utilizing HIVST.

*Most of the time I think of the test outcomes […] what if the test shows that I am [HIV] positive? […] What will I do? […] I like to be tested by someone else so that if I am positive and the service provider is friendly, then she will know how to make me feel like a normal person***[IDI_21 years_Mbeya]**

*When people discover that they have HIV, they always get shocked. Having HIV may cause people to commit suicide. So, it’s a hundred times better if the test is done by a trained person who provides counselling so that persons who test HIV positive come to term with their condition***[IDI_41 years_Iringa]**

Multiple participants from PGDs and IDIs were also concerned about users’ error, especially relating to lack of capacity to interpret the test among FSWs. Some participants were sceptical about the credibility an individual would put in a result obtained by oneself. One FSW participating in IDI expressed a Kiswahili saying “*mganga hajigangi*” (a healer cannot heal / treat her / himself), to describe how even if FSWs were trained, they could not diagnose themselves.

*There is a Kiswahili proverb that says, a doctor cannot diagnose or heal her/himself. (Laughter) We don’t have the courage and skills to test ourselves […] You may test and deceive yourself that you have tested negative while you are HIV positive***[IDI_32 years_Mbeya]**

In two PGD sessions, participants raised the concern that HIVST might damage or strain marital relationships. Some participants wondered that availability of HIVST kits might encourage male partners to force their spouses to test and disclose her status. The main concern was the potential for physical harm or psychological distress in case of discordant HIV results within a couple.

## Discussion

HIVST is a new approach for HIV testing, and at the time we conducted this study, HIVST had not been included in the Tanzania comprehensive national HIV control package [45]. Although the majority of study participants lacked the experience of using HIVST, they demonstrated great enthusiasm towards using the approach once available. A belief that HIVST will increase privacy and confidentiality were key factors that motivated FSWs towards HIVST. Studies on facility-based HTS conducted in SSA have associated low uptake with concerns about a breach of confidentiality [46] and fear of HIV stigma [47]. Participants in our study described their ‘lived experience’ of stigma and the societal perceptions portrayed towards FSWs as drivers of HIV transmissions. This discrimination impacts health care seeking, as documented in a study where FSWs reported to be treated poorly by health care providers [48]. In our study, participants related stigma to both fatalistic attitudes (i.e. perceptions that FSWs have HIV) and a feeling that they did not want to test. In Kenya, persons with high-risk profiles were less likely to accept HIVST than those with low-risk profiles [49]. These attitudes can negatively impact the rollout of HIVST. Thus, programs need to address these widely shared attitudes, as well as stigma and other structural barriers to HIV care at an individual level.

Participants in our study believed that the introduction of HIVST would bring a major change in the HTS landscape by minimizing dependence on health workers and saving time and money on testing. The concerns about direct and opportunity costs have been echoed by a study which looked at obstacles to syphilis diagnosis and treatment in Tanzania [50]. A study in South Africa showed that introduction of HIVST resulted in increased frequency of testing among men who have sex with men (MSM) [51]. Besides, a study conducted among FSWs in Zambia and Uganda showed that HIVST has the potential to increase uptake of other HIV prevention interventions such as PrEP [52]. This makes programmers optimistic that it could similarly occur in Tanzania after the introduction of the service.

Enthusiasm toward HIVST came along with concerns about potential self-harm or harm from others in case of a reactive HIV test. The most prominent concern was that HIVST could cause adverse social outcomes, including psychological distress, partner violence and even suicide. This was linked to the study participants’ perception that HIVST would be provided only through private / unassisted modality. The fear of potential social and psychological harm related to the absence of pre-testing counselling has also been reported in Kenya and South Africa [53, 54]. However, this fear may be more of a perception than a reality since a recent systematic review did not find any strong evidence that HIVST leads to serious social harm (partner violence and suicides) [55]. Nevertheless, any risk of harm must be taken seriously. Thus, the rollout of HIVST among FSWs should follow all current evidence and guidelines to reduce the risk of harm, keeping in consideration their increased risk associated with societal norms. To achieve success with this novel approach, incorporating user perspectives is essential. Furthermore, research on partnerships, communication, discordance, and health care seeking choices for FSWs in relation to HIVST may help clarify the best way to roll out HIVST.

In this study, some participants were concerned about both the accuracy of test results as well as the user’s ability to interpret results. User errors were perceived to be particularly problematic for FSWs given low literacy levels. These views were not unique to participants in the current study: misgivings about user error with HIVST has also been reported by other key populations in studies across SSA and beyond [36, 51]. However, a recent systematic review on HIVST has shown that laypersons can perform HIVST accurately without or with little help or supervision of health care providers [35]; even though, authors from that study cautioned that accuracy in reading the test must be closely monitored [35]. Our study supports that note of caution.

### Limitations

This study should be viewed in light of some limitations. Since at the time we conducted this study HIVST had not yet been introduced in Tanzania, the knowledge about the self-testing approach was theoretical. Therefore, it is not clear to what extent these views would translate into real health care choices by FSWs’ populations. Since some FSWs in the study had already been exposed to the combination HIV prevention interventions by Sauti program in the study regions, they may have provided what they thought were socially desirable responses, particularly about the uptake of HIV testing. The study sample for this study was small, which limits the generalizability of the study to the larger FSWs population in Tanzania. Therefore, additional research into lived experiences of FSWs using HIVST would be beneficial to build on the findings of this and other exploratory studies.

## Conclusion

Our study results indicate strong support for the use of HIVST by FSWs if made available in Tanzania, for reasons of convenience (time and transport-related cost that will be saved) and increased confidentiality. Enthusiasm toward HIVST went alongside concerns about potential self-harm or harm from others in case of reactive test, as well as apprehension about FSWs ability to interpret test results, in light of low literacy among this population. With the right support in place, there is a strong possibility that HIVST will be an appropriate approach for HIV testing among FSWs as a population at high risk of HIV infection but with limited access to health services in Tanzania. However, since this study was conducted to explore the feasibility of introducing HIVST, additional research into lived experiences of FSWs using HIVST would be beneficial to build on the findings of this and other exploratory studies.

## Data Availability

Permission to publish data from this study was granted by the Medical Research Coordinating Committee of the National Institute for Medical Research (NIMR) and by the Johns Hopkins Bloomberg School of Public Health. Upon reasonable request, the dataset files used for the analysis and preparation of this study are accessible at the National Institute for Medical Research (NIMR) and from the Sauti Program, Jhpiego Tanzania - an affiliate of Johns Hopkins University.

## Abbreviations

CBOs: Community Based Organisations
CSOs: Civil Society Organisations
FSW(s): Female Sex Worker(s)
HIV: Human Immunodeficiency Virus
HIVST: HIV Self Testing
HTS: HIV testing services
IDI: In-Depth Interview
KP: Key Populations (for HIV infection)
MoHCDGEC: Ministry of Health, Community Dev., Gender, Elderly and Children
MSEM: Modified Social-Ecological Model
MSM: Men who have sex with men
NIMR: National Institute for Medical Research
PGD: Participatory Group Discussions
PrEP: Pre-Exposure Prophylaxis
SEM: Socio-Ecological Model
SSA: Sub-Saharan Africa
SOP: Standard operating procedure
USAID: US Agency for International Development
WHO: World Health Organization
